# Copy number variation of *IL17RA* gene and its association with the ankylosing spondylitis risk in Iranian patients: a case-control study

**DOI:** 10.1186/s12881-020-01078-y

**Published:** 2020-07-10

**Authors:** Hamideh Aghaei, Elham Farhadi, Maryam Akhtari, Sara Shahba, Shayan Mostafaei, Ahmadreza Jamshidi, Shiva Poursani, Mahdi Mahmoudi, Mohammad Hossein Nicknam

**Affiliations:** 1 Department of Immunology, School of Medicine, Tehran University of Medical Sciences, Tehran, Iran; 2 Rheumatology Research Center, Tehran University of Medical Sciences, Tehran, Iran; 3 Inflammation Research Center, Tehran University of Medical Sciences, Tehran, Iran; 4 Department of Biostatistics, School of Health, Kermanshah University of Medical Sciences, Kermanshah, Iran; 5 Molecular Immunology Research Center, Tehran University of Medical Sciences, Tehran, Iran

**Keywords:** Ankylosing spondylitis, Autoimmunity, Copy number variations, Gene expression, IL17RA

## Abstract

**Background:**

Ankylosing spondylitis (AS) is considered as a subtype of spondyloarthritis (SpA) that mainly leads to fatigue, stiffness, spinal ankylosis, and impaired physical functions with reduced quality of life. Interleukin (IL)-17A provokes additional inflammatory mediators and recruits immune cells to the inflamed site. *IL17* expression increased in various inflammatory disorders including psoriasis, rheumatoid arthritis, multiple sclerosis, crohn’s disease, and ankylosing spondylitis. The current study aimed to evaluate the association of *IL17RA* copy number changes with the susceptibility to AS and their correlation to *IL17RA* expression in Iranian population.

**Methods:**

*IL17RA* copy number genotyping assessments were carried out in 455 AS patients and 450 healthy controls, using custom TaqMan CNV assays. TaqMan primers and probe were located in Chr.22:17109553 based on pre-designed *IL17RA* Copy Number Assay ID, Hs02339506_cn. mRNA expression of *IL17RA* was also measured by SYBR Green real-time polymerase chain reaction (PCR).

**Results:**

A *IL17RA* copy number loss (< 2) was associated with AS compared to 2 copies as reference (OR:2.18, 95% CI: (1.38–3.44), *P*-value < 0.001) and increased the risk of AS. *IL17RA* mRNA expression showed a significant increase in peripheral blood mononuclear cells (PBMCs) of all AS individuals than controls. The mRNA expression level of 2 copies was significantly higher in AS patients.

**Conclusions:**

Our findings revealed that a low copy number of *IL17RA* might confer a susceptibility risk to AS. However, it is probably not directly involved in the regulation of *IL17RA* mRNA expression. Epigenetic mechanisms like DNA methylation, post-transcriptional, and -translational modifications that regulate the expression of the genes may contribute in upregulation of *IL17RA* mRNA expression in the loss of gene copy number condition.

## Background

Ankylosing spondylitis (AS) is considered as a subtype of spondyloarthritis (SpA) that mainly leads to fatigue, stiffness, spinal ankylosis, and impaired physical functions with reduced quality of life (QoL) [1, 2]. In spite of various investigations concerning AS pathogenesis, the exact cause, and underlying mechanisms are still less clear [3–6]. However, according to the numerous studies taken in the past decades, genetic background and immunological dysfunctions are regarded as pivotal bases of the disease [7–10].

Interleukin (IL)-17A, the first known subset of the *IL17* family, primarily secreted from multiple cell types including, γδ T cells, natural killer T cells (NKT cells), group 3 Innate lymphoid cells (ILC3s) and particularly Th17 [11, 12]. This pro-inflammatory cytokine provokes additional inflammatory mediators and recruits immune cells to the inflamed tissue. IL-17 mostly leads to production of several chemical messengers such as proinflammatory cytokines (IL-6, IL-8, TNF-α, IL-1β), granulocyte attracting chemokines (granulocyte-colony stimulating factor (G-CSF), granulocyte monocyte-colony stimulating factor (GM-CSF), CCL2 (MCP-1), CXCL2 (MIP-2), CXCL5, CCL20 (MIP-3A)), matrix metalloproteinases (MMP1, MMP3, MMP9, and MMP13) and anti-microbial peptides [13–16].

Taken together, the aberrant IL-17 signaling pathway contribute to developing several autoimmune disorders [12]. It is clearly stated that the excessive production of IL-17 can lead to various inflammatory diseases such as psoriasis, rheumatoid arthritis (RA), AS, multiple sclerosis (MS) and crohn’s disease (CD) [17–19]. Accordingly, prior investigations have found the increased concentration of IL-17 in the serum of AS patients and the frequency of Th17 cells also elevated in peripheral blood and synovial fluids of AS patients [20, 21].

The IL-17 activation is provided after binding to receptor site on target cells. As previously reported, IL-17 binds to a complex of IL-17RA and IL-17RC to induce responses via its unique signaling pathway [22]. IL-17RA (also termed as IL-17R), is the first member of IL-17R superfamily (IL-17RA-IL-17RE) which recognizes both IL-17A and IL-17F, but have more affinity to IL-17A than IL-17F about 100 fold greater [23]. This family is distinctive from other known cytokine receptor families with its particular structure and signaling cascade through ACT1 (NF-κB activator 1) adaptor molecule [24]. *IL17RA* showed expression on the variety of tissues, and cell types, particularly in hematopoietic cells and upon stimulation with its ligand can activate NF-κB, a major inflammatory transcription factor [25, 26]. In addition to its capacity to induce unbalanced cytokine production, it can reinforce the effect of other potent cytokines including TNF-α and IL-1β, which leads to prolonged inflammatory responses and exacerbate chronic inflammations [27, 28].

To date, there are mysteries in genetic aspects of AS. Recent discoveries have emphasized the substantial role of single nucleotide polymorphisms (SNPs) in AS pathogenesis [29–32]. The newly performed study by Vidal-Castiñeira et al. assessed the association of *IL17RA* polymorphisms with AS risk [33]. Besides SNPs, copy number variations (CNVs) are believed to contribute to the etiology of ankylosing spondylitis. They are known as different copies of a specific fragment of DNA created due to deletions or duplications that captured 1 Kilobase to several megabases of the genome [34]. Given that CNVs are harboring 12% of the human genome, they can strongly affect the expression of genes encoding proteins [35]. CNVs exert their influence on gene expression through gene dosage imbalances in dosage-sensitive genes and cause changes in mRNA and eventually in protein level [36]. In addition to simple gene dosage effect, rearrangements in the gene regulatory elements can also alter gene expression. These rearrangements lead to changes in the interval between genes to regulatory regions. New fused genes can form as a result of rearrangements [37]. Moreover, a growing number of reports underscore the importance of CNVs in some complex diseases such as RA, psoriasis, Crohn’s disease, and systemic lupus erythematosus (SLE), autism, schizophrenia, type 1 diabetes (T1D), human immunodeficiency virus (HIV) and cancers [38, 39].

Recently, a genome-wide copy number variation analysis has identified 9 loci as potential candidate CNV regions [40]. Among them, IL17RA has been significantly associated with AS and showed a protective role in susceptibility to AS [40]. To gain more insight into the genetic structure of AS, it would be instrumental in following up initial clues provided by the copy number variation analysis study.

With regards to *IL17RA* expression on immune cells, it seems that changes in the copy number of the gene might influence the gene by altering its expression. However, the contribution of *IL17RA* gene CNVs in the pathogenesis of AS and its influence on *IL17RA* gene expression has not been investigated yet. Here, we set out to determine whether *IL17RA* copy number variations would predispose individuals to the AS in the Iranian population.

## Methods

### Study populations

A total of 455 AS patients (85.1% males) and 450 age-, race and sex-matched healthy controls (87.3% males) were involved. All AS patients were recruited from the rheumatology clinic, Shariati Hospital, Tehran, and met the modified New York diagnostic criteria for AS [41]. All controls had no family history of AS or other autoimmune diseases. More detailed characteristics of enrolled participants are represented in Table 1. The written informed consent was signed by all members of the case and control groups before enrolling in the study. Moreover, this study was approved by ethics committee at the Tehran University of Medical Sciences (Approval ID: IR.TUMS.MEDICINE.REC.1398.696).

**Table 1:** Demographic data and clinical features of AS patients and healthy controls recruited in this study

Characteristics	AS patients ***N*** = 455 (%)	HCs ***N*** = 450(%)	***P***-value
**Sex (Male)**	387 (85.1%)	393 (87.3%)	0.37
**Age**	38.10 ± 10.46	36.60 ± 8.25	0.86
**ESR**	20.28 ± 20.19	5.84 ± 6.18	< 0.001***
**Age symptom**	23.38 ± 8.43	–	NA
**Age diagnosis**	30.93 ± 9.45	–	NA
**Disease duration**	14.88 ± 9.37	–	NA
**NRSBASG**	9.73 ± 5.36	–	NA
**ASQOL**	7.67 ± 5.26	–	NA
**BASMI**	4.07 ± 1.86	–	NA
**BASDAI**	4.72 ± 2.48	–	NA
**BASFI**	3.82 ± 2.59	–	NA

All values are represented as Mean ± Standard Deviation; *NA* not applicable, *AS* ankylosing spondylitis, *HCs* healthy controls, *ESR* erythrocyte sedimentation rate, *NRS-BASG* numerical rating scale-bath AS patient global score, *ASQOL* ankylosing spondylitis quality of life, *BASMI* bath ankylosing spondylitis metrology index, *BASDAI* bath ankylosing spondylitis disease activity index, *BASFI* bath ankylosing spondylitis functional index

* *P* < 0.05; ** *P* < 0.01; *** *P* < 0.001

### Genomic DNA extraction

The blood sample was taken from participants, and the isolation of peripheral blood mononuclear cells (PBMCs) were done based on Ficoll-Hypaque gradient technique (innotrain, Germany). DNA samples were isolated from PBMCs in standard collection tube with EDTA as anticoagulants using DNSol MidiPrep DNA extraction kit (Roje Technologies Co., Iran). For evaluating the purity of DNA, the absorbance of DNA samples was measured at 260 and 280 nm using Thermo Scientific/2000C Nanodrop. Pure DNA has an A260/A280 ratio ranges from 1.8–2.0. Extracted DNA from all samples were stored at − 20 °C until copy number evaluation.

### Determination of the *IL17RA* copy number

**Fig. 1:**
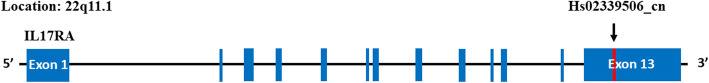
The genetic structure of *IL17RA* gene and the position of Hs02339506_cm TaqMan assay containing primers and probe

For analysis of *IL17RA* gene copy number, we used the predesigned TaqMan CNV qPCR assay (Assay ID: Hs02339506_cn), which is purchased from Applied Biosystems (Foster City, CA, USA) (http://bioinfo.appliedbiosystems.com/genomedatabase/copy-number-variation.html). *IL17RA* TaqMan assay includes 2 target-specific primers and a FAM/MGB dual-labeled probes which are located in Chr.22:17109553 (within exon 13). The genetic structure of *IL17RA* gene and the position of Hs02339506_cm TaqMan assay containing primer sets and probe illustrated in Fig. 1. Additionally, TaqMan Copy Number Reference Assay (RNase P) includes two primers and VIC/TAMRA labeled probes which used as a standard reference assay recommended by the manufacturer’s protocol. Each reaction included 5 μl of 2× TaqMan Genotyping Master Mix, 0.5 μl of *IL17RA* TaqMan copy number assay, 0.5 μl of TaqMan Copy Number RNAse P Reference Assay, 2.5 μl of water, and 1.5 μl of 10 ng/μl genomic DNA in a 10 μl total volume. All samples were run in duplicate in a 96 well plate, and fluorescence signals emitting from reporter dyes (FAM and VIC) were normalized to ROX (passive reference dye). Real-time PCR was done under thermal cycling program comprised of 1 holding stage of 10 min at 95 °C, followed by 40 cycles in 15 s at 95 °C and 60 s at 60 °C.

Amplification data were analyzed using Applied Biosystems StepOnePlus software (version 2.3), and raw data of CT (threshold cycle) values were imported to CopyCaller® Software v2.0 (Applied Biosystems) to calculate the gene copy number in each sample. The comparative Ct method (ΔΔCt formula) was applied to estimate the relative gene copy number. To grant validity to results, confidence values were higher than 95%, and Z-Score was < 1.75. Besides, CT of the reference gene was set < 32, and the SD of ΔCT between replicates was less than ±0.2.

### mRNA expression of *IL17RA*

First, total RNA extraction was implemented from PBMCs of 68 cases and 53 controls with the High Pure RNA isolation kit (Roche, Germany). Total RNA concentration was determined by measuring the absorbance at 260 nm in Thermo Scientific/2000C Nanodrop. Afterward, reverse transcription (RT) was performed to synthesize cDNA from RNA by the PrimeScript RT reagent Kit (Takara, Japan). Subsequently, relative quantification of mRNA expression was conducted by SYBR Green qPCR on a StepOnePlus Real-time PCR system. Gene expression analysis was carried out with specific primers for *IL17RA* (Forward primer: 5′-AGTTCCACCAGCGATCCAAC-3′; Reverse primer: 5′-GTCTGAGGCAGTCATTGAGGC-3′) and for β2-microglobulin (*β2M*) (Forward primer: 5′-CCTGAATTGCTATGTGTCTGGG-3′; Reverse primer: 5′- TGATGCTGCTTACATGTCTCGA-3′). The mRNA expression was normalized to *β2M*.

### Data analysis

The Kolmogorov–Smirnov test was implemented for computing normality of tests for continuous variables. We performed Mann-Whitney U non-parametric test to compare the gene expression data between the two groups. Correlation analysis was done by Spearman’s rank correlation. Pearson Chi-square test was used to assess the association between the categorical variables.

Furthermore, for checking the effect size odds ratio (OR) with 95% confidence intervals (95% CI) was measured. All data were analyzed using IBM SPSS version 21.0 (SPSS, Chicago, IL, USA). In addition, experimental data graphs were designed by GraphPad Prism version 6 (La Jolla, CA, USA). Results with *P* value less than 0.05 was regarded to be statistically significant.

## Results

### Characteristics of study subjects

The demographic and clinical features of studied subjects are listed in Table 1. The mean ± SD age of patients and healthy individuals were 38.10 ± 10.46 and 36.60 ± 8.25 years, respectively. There was no statistically significant difference between two groups concerning sex (*P*-value = 0.37) and age (*P*-value = 0.86). Patients with AS had higher levels of serum ESR compared to controls (*P*-value < 0.001).

### Analysis of *IL17RA* CNV

Based on our results, *IL17RA* copy number varied from 1 to 4 per diploid genome. As shown in Table 2, CN = 2 was the predominant copy count in both groups. One copy number of *IL17RA* describe gene deletion, while 3 and 4 copy counts are indicative of gene duplication relative to normal 2 copy number. The frequency of two copies of *IL17RA* was 76.3% in AS subjects and 85.6% in the control group. 13.4% of cases and 6.9% of controls exhibited l copy (CN <  2) of *IL17RA* and were remarkably significant. A high copy of CN seemed to be more in AS patients comparing to healthy subjects (10.3% vs. 7.6%), but the difference was not significant. Only 2 patients possessed 4 copies. Based on the results of chi-square test, the overall distribution of the copy-number in the cases compare to control was significantly different (*P*-value = 0.001).

**Table 2:** Frequency distribution of *IL17RA* copy numbers in cases and controls and estimation of AS risk. (N (%))

Copy Number of *IL****17RA***	AS (N = 455)	HCs (N = 450)	OR (95% CI)	***P*** value
**=2 Copies**	347 (76.3%)	385 (85.6%)	Ref.	Ref.
**< 2 Copies**	61 (13.4%)	31 (6.9%)	2.18 (1.38–3.44)	< 0.001^a^*
**> 2 Copies**	47 (10.3%)	34 (7.6%)	1.53 (0.963–2.44)	0.071 ^a^

*AS* ankylosing spondylitis, *HCs* healthy controls.

^a^Compared with = 2 copies

*OR* Odds ratios, *95% CI* 95% confidence interval.

*P value < 0.05 has statistical significance

Copy numbers were categorized into 3 groups as following: CN < 1 (losses), CN = 2 (neutral), and CN >  2 (gains). As shown in Table 2, the frequency of CN loss was significantly higher in AS patients (vs. 2 copies OR:2.18, 95% CI: (1.38–3.44), *P*-value < 0.001) in comparison to controls. Although there was no significant difference between the two groups for CNV gain (vs. 2 copies OR:1.53, 95% CI: (0.963–2.44), *P*-value = 0.071). Of note, two copies considered as the reference for estimation of AS risk.

### *IL17RA* mRNA expression level

**Fig. 2:**
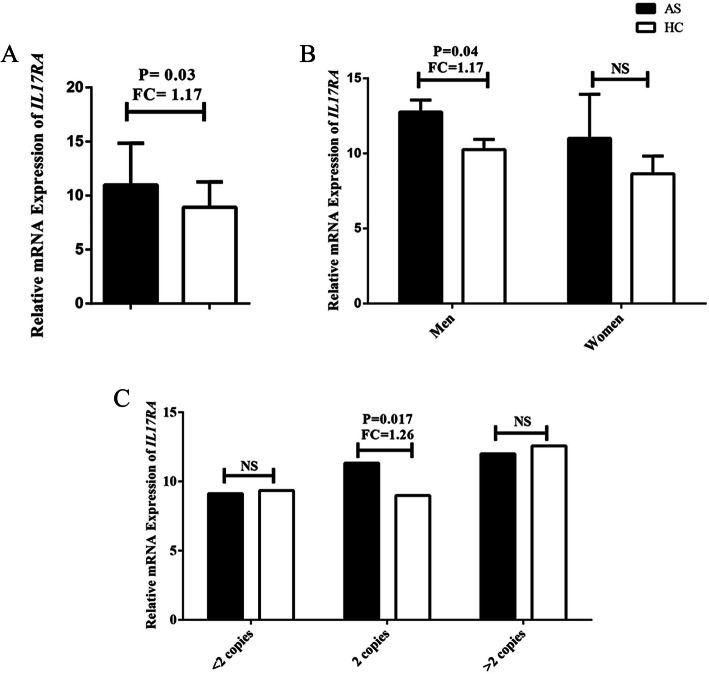
**a** Relative gene expression of *IL17RA* in all AS patients and healthy controls (HC). **b** Relative gene expression of *IL17RA* in AS and healthy control groups adjusted by sex. **c** Relative gene expression of *IL17RA* in AS and healthy control groups adjusted by *IL17RA* copy numbers, NS, not significant

To address the association of *IL17RA* CN to the expression level of this gene, we analyzed *IL17RA* expression in PBMCs of 68 cases and 53 controls. The expression of *IL17RA* was higher in all cases compared to controls (*P*-value = 0.032, Fold change: AS/Control = 1.17) (Fig. 2a). Interestingly, the mRNA level displayed a significant raise in men compared to women. The detailed data are shown in Table 3 and Fig. 2b. The mRNA expression was also assessed by different copy numbers status (Table 4 and Fig. 2c). *IL17RA* mRNA expression fold change elevated in AS patients in 2 copies group. In contrast, there were no significant mRNA expression changes in CN loss and CN gain groups.

**Table 3:** *IL17RA* mRNA expression in AS and healthy control groups adjusted by sex (Median ± Standard Deviation)

The mRNA expression level of ***IL17RA***	AS (N = 68)	HCs (***N*** = 53)	Fold Change (AS/ HC)	***P***-value
**Overall**	10.73 ± 5.82	9.19 ± 4.62	1.17	0.032*
**Men**	10.81 ± 5.81	9.26 ± 4.75	1.17	0.041*
**Women**	9.79 ± 6.57	8.38 ± 2.37	1.17	0.988

*AS* ankylosing spondylitis, *HCs* healthy controls.

*P-value < 0.05 has statistical significance

**Table 4:** *IL17RA* mRNA expression in AS and healthy control groups adjusted by the copy number of *IL17RA*

Copy Number of ***IL17RA***	AS (N = 68)	Control (N = 53)	Fold Change (AS/ HC)	***P*** value
**=2 Copies**	11.33 ± 6.40	8.99 ± 4.65	1.26	0.017*
**< 2 Copies**	9.12 ± 3.89	9.34 ± 3.15	0.976	0.969
**> 2 Copies**	12.0 ± 5.83	12.57 ± 6.13	0.954	0.843

*P value < 0.05 has statistical significance

### Correlation between copy number and mRNA expression of *IL17RA* with clinical features of AS

As shown in Table 5, there was no correlation between each of *IL17RA* copy number categories and clinical characteristics in AS patients. Moreover, *IL17RA* mRNA expression was not correlated significantly with clinical data (Table 5). However, BASFI and BASMI have higher non-significant effects on *IL17RA* mRNA expression (Table 5).

**Table 5:** Associations between mRNA expression and copy number of *IL17RA* with clinical features of AS

AS patients (***N*** = 68)							
Clinical features		ESR, mm/h 24 ± 22.09	Disease duration, years 9.47 ± 9.72	BASDAI score 5.20 ± 2.61	BASFI score 3.76 ± 2.43	BASMI score 3.43 ± 1.69	ASQol score 8.29 ± 5.36
***IL17RA***							
**mRNA expression**	***r***	0.026	0.015	−0.032	0.111	0.133	0.045
	***P***	0.78	0.91	0.81	0.40	0.34	0.74
**Copy number**	***r***	−0.102	−0.016	0.174	0.134	0.248	0.112
	***P***	0.28	0.90	0.18	0.30	0.07	0.40
**mRNA expression**	***β (SE)***	0.08 (0.06)	0.23 (0.19)	−0.84 (0.69)	1.19 (0.83)	−1.2 (0.85)	−0.17 (0.34)
	***P***	0.19	0.25	0.23	0.16	0.17	0.62

*BASFI* bath ankylosing spondylitis functional index, *BASDAI* bath ankylosing spondylitis disease activity index, *BASMI* bath ankylosing spondylitis metrology index, *ASQOL* ankylosing spondylitis quality of life, *P* Adjusted *P*-value by Benjamini and Hochberg method to control false discovery rate in multiple testing, *SE* standard error of coefficient (β), r: Spearman’s correlation coefficient

## Discussion

In the present study, the *IL17RA* CNVs and mRNA expression levels were evaluated to provide more information about its role in AS disease risk and IL-17A inflammatory pathways.

The fundamental role of IL-17A pathway in AS pathogenesis was identified by the high frequency of Th17 cells and IL-17A expression level in the peripheral blood of AS patients. Several studies showed the importance of IL-17A as a major cytokine that mediates responses in inflammation, infection, and autoimmune diseases through binding to its unique receptor (IL-17RA) and stimulates ACT1 signaling [42]. IL-17RA signaling pathway contributes to the trigger expression of several inflammatory mediators in the active phase of inflammatory diseases [43].

In the recent years, the genome-wide association studies (GWASs) showed the contribution of genes outside of the MHC region in the risk of AS and CNVs received more attention due to their pathophysiological role in autoimmune diseases [44]. A GWAS carried out by Jung et al. has determined 9 candidate genes as CNVs regions related to AS risk in the Korean population. Among other genes, the only gain in *IL17RA* was significantly protective against AS, and its influence is suggested in AS development [40]. Recently, Shahba et al. reported the association signal between *BMP8A* copy number variation and susceptibility to AS in Iranian patients [45]**.**

In line with Korean study [40], we showed that having only less than 2 copies of *IL17RA* gene significantly increased susceptibility to AS. Therefore, it suggests that there is a direct association between low copy number and AS development in both populations. These results proposed that loss of *IL17RA* is considered as an effective CNV in AS development in Iranian population. When stratified for sex, no sex bias was detected in CNVs categories in cases and controls. Additionally, for the first time, we examined *IL17RA* mRNA expression level to understand how the CNVs of the *IL17RA* can affect expression of the gene.

Despite the fact, we have observed the loss of gene copy number associated with disease susceptibility, and there was no significant change in mRNA expression level in cases and controls with less than 2 copies. While the increased expression of *IL17RA* mRNA has been observed in 2 copies group.

Therefore, a lack of significant association of *IL17RA* CNVs with mRNA expression levels in both groups could be attributed to epigenetic factors and modifications, dysfunction of a transcription factor, and also a specific mutation that may contribute to *IL17RA* upregulation in AS [46]. DNA methylation, as one of the most epigenetic modifications, has been recognized to influence gene expression [47]. All these strategies can naturally occur in an intuitive phenomenon called genetic compensation. It is a regulatory process that fundamentally responds to the abnormal changes in RNA or protein level [48].

Furthermore, it is expressed that the phenotypic effects of CNVs may have not a direct relationship to gene expression. Some biological mechanisms are proposed to make different phenotypes associated with upregulation and dysregulation of immune responses such as position effect, extra protein-coding domains, and so on [49, 50]. PBMCs isolation is a less invasive alternative method for measuring expression level as well as copy number of genes compared to the synovial biopsy isolation, therefore, we evaluated the *IL17RA* expression in PBMCs. The results that no significant change between the different copy number category of *IL17RA* gene and its mRNA level might not be true for other tissues, as the expression of this gene varies from tissue to tissue. However, a recent study has studied the genes expression profile in PBMCs of AS patients with inflamed synovial tissue. The authors found a number of immune-associated genes with similar expression change in both PBMCs and synovial tissues. This study suggested that local inflammation could show a reflection in PBMCs alterations [51].

Because of the possible importance of this cytokine in AS and similar conditions, as we expected, the mRNA expression level of *IL17RA* was significantly higher in all our patients compared to controls. Previous studies indicated increased serum IL-17 level in AS patients showing that this cytokine may play a crucial role with IL-23 in the AS pathogenesis [20, 52, 53]. Following our findings, *IL17RA* expression was also significantly higher in synoviocytes of RA and psoriatic arthritis (PsA) patients [54]. Reduced production of proinflammatory mediators (e.g., IL-1, IL-6, and different MMPs) along with lessened joint inflammation has been shown in mice with IL17R-deficient streptococcal cell wall-induced arthritis [55].

Therefore, the upregulation of *IL17RA* could enhance their response to IL-17A and exacerbate inflammation of the joints [43]. In accordance with the previous studies and the current research, it is highly possible to consider an essential role for the IL-17 family with corresponding receptors in ankylosing spondylitis.

The genetic architecture, which is an essential determinant of AS, making it a polygenic complex disease with a more heritable tendency to affect males than females [56, 57]. In our study, increased *IL17RA* mRNA expression is restricted to male patients, after adjusting by sex. Consistent with our results, Gracey et al. found the upregulation of *IL17RA* expression in male AS patients [58]. Furthermore, there are not any significant correlation between CNVs and mRNA expression level with demographic and clinical data of patients in our study.

## Conclusion

In conclusion, regarding the frequency distribution of copy numbers, less than 2 copies of *IL17RA* were significantly susceptible to AS in this cohort of Iranian patients. Epigenetic mechanisms like DNA methylation, post-transcriptional, and -translational modifications that regulate the expression of the genes may contribute in upregulation of *IL17RA* mRNA expression in loss of the gene copy number. On the other hand, the significant upregulation mRNA transcript level of *IL17RA* in all AS patients indicates the critical role of this pathway in the risk of AS. Further researches, especially in the context of functional consequences, seem to be necessary to illuminate the more details of the fundamental role of CNVs with biologic effects.

## Supplementary information

**Additional file 1: Supplementary file 1(.xlsx).** The data of mRNA expression and copy number of *IL17RA* in studied patients and controls.

## Data Availability

The relevant raw data of this study are available as an Additional file 1.

## Abbreviations

CNV: Copy number variation
CD: Crohn’s disease
GWAS: Genome-wide association study
GM-CSF: Granulocyte monocyte-colony stimulating factor
G-CSF: Granulocyte-colony stimulating factor
HIV: Human immunodeficiency virus
ILC3: Innate lymphoid cell
IL: Interleukin
MMP: Matrix metalloproteinases
MS: Multiple sclerosis
NKT cells: Natural killer T cells
PsA: Psoriatic arthritis
QoL: Quality of life
RT: Reverse transcription
RA: Rheumatoid arthritis
SNP: Single nucleotide polymorphism
SpA: Spondyloarthritis
SLE: Systemic lupus erythematosus
T1D: Type 1 diabetes
Β2M: β2-microglobulin;
